# A comparison of LKB1/AMPK/mTOR metabolic axis response to global ischaemia in brain, heart, liver and kidney in a rat model of cardiac arrest

**DOI:** 10.1186/s12860-018-0159-y

**Published:** 2018-06-19

**Authors:** Shohreh Majd, John H. T. Power, Timothy K. Chataway, Hugh J. M. Grantham

**Affiliations:** 1 Centre for Neuroscience, Neuronal Injury and Repair Laboratory, College of Medicine and Health Sciences, Flinders University, Adelaide, SA 5042 Australia; 2 Centre for Neuroscience, Neuronal Injury and Repair Laboratory, College of Medicine and Public Health, Flinders University of South Australia, Adelaide, SA 5042 Australia; 3 Proteomics Facility, College of Medicine and Public Health, Flinders University, Adelaide, SA 5042 Australia

**Keywords:** Cell energy stress, ADP/ATP, Ischaemia, Liver kinase b1, LKB, Adenosine monophosphate kinase protein kinase, AMPK, Mammalian target of rapamycin, mTOR, Brain, Heart, Liver, Kidney, Cardiac arrest, Sprague Dawley rat, Western blot, Bioluminescent assay

## Abstract

**Background:**

Cellular energy failure in high metabolic rate organs is one of the underlying causes for many disorders such as neurodegenerative diseases, cardiomyopathies, liver and renal failures. In the past decade, numerous studies have discovered the cellular axis of LKB1/AMPK/mTOR as an essential modulator of cell homeostasis in response to energy stress. Through regulating adaptive mechanisms, this axis adjusts the energy availability to its demand by a systematized control on metabolism. Energy stress, however, could be sensed at different levels in various tissues, leading to applying different strategies in response to hypoxic insults.

**Methods:**

Here the immediate strategies of high metabolic rate organs to time-dependent short episodes of ischaemia were studied by using a rat model (*n* = 6/group) of cardiac arrest (CA) (15 and 30 s, 1, 2, 4 and 8 min CA). Using western blot analysis, we examined the responses of LKB1/AMPK/mTOR pathway in brain, heart, liver and kidney from 15 s up to 8 min of global ischaemia. The ratio of ADP/ATP was assessed in all ischemic and control groups, using ApoSENSOR bioluminescent assay kit.

**Results:**

Brain, followed by kidney showed the early dephosphorylation response in AMPK (Thr^172^) and LKB1 (Ser^431^); in the absence of ATP decline (ADP/ATP elevation). Dephosphorylation of AMPK was followed by rephosphorylation and hyperphosphorylation, which was associated with a significant ATP decline. While heart’s activity of AMPK and LKB1 remained at the same level during short episodes of ischaemia, liver’s LKB1 was dephosphorylated after 2 min. AMPK response to ischaemia in liver was mainly based on an early alternative and a late constant hyperphosphorylation. No significant changes was observed in mTOR activity in all groups.

**Conclusion:**

Together our results suggest that early AMPK dephosphorylation followed by late hyperphosphorylation is the strategy of brain and kidney in response to ischaemia. While the liver seemed to get benefit of its AMPK system in early ischameia, possibly to stabilize ATP, the level of LKB1/AMPK activity in heart remained unchanged in short ischaemic episodes up to 8 min. Further researches must be conducted to elucidate the molecular mechanism underlying LKB1/AMPK response to oxygen supply.

## Background

Under conditions of energy stress, it is critical for all tissues to adjust their metabolic demands to their energy supplies [1]. During metabolic stress situations, adenosine monophosphate protein kinase protein (AMPK) acts as the master of cell energy regulator, establishing homeostasis via shutting off the ATP consuming anabolic pathways while it switches on the catabolic pathways, producing ATP [2, 3]. AMPK is a highly conserved serine/threonine protein kinase composed of a catalytic α-subunit and two regulatory β and γ-subunits. Previous studies demonstrated that metabolic stress, which increased the ratio of ADP, and consequently AMP to ATP, activated AMPK α-subunit to more than 100 folds through its phosphorylation at Threonine 172 (Thr^172^) [4, 5]. One of the main kinases to mediate AMPK phosphorylation is liver kinase b1 (LKB1), originally was introduced as a tumor suppressor enzyme [6, 7]. LKB1 is believed to be constitutively active, however its higher phosphorylation at Serine 431 (Ser^431^) in response to some stimuli such as ischaemia, increases the activation of these kinase. This causes it to phosphorylate AMPK more rapidly if AMP binds to AMPK γ-subunit [8, 9]. Apart from LKB1/AMPK role in cell energy homeostasis, they contribute to cell proliferation, cell polarity regulation, gene transcription and cellular growth [10, 11]. A substantial part of this regulatory role is applied through mammalian target of rapamycin (mTOR), one of AMPK targets, with many suggested roles in cell metabolism, growth and proliferation [12]. Phosphorylation of AMPK at Thr^172^ inhibits mTOR activity and consequently cell growth and proliferation. That assists cells in putting a hold on using energy resources for cell proliferation and growth during energy stress [13]. AMPK activation is also mediated by reactive oxygen spices (ROS) and calcium calmodulin in an independent way to ADP/ATP ratio [14, 15]; however the rational of cell preference in choosing the mechanism of AMPK activation under different circumstances is still under investigation.

Hypoxic damage particularly to high metabolic rate tissues is one of the main underlying causes for the organs’ failure due to many factors such as initial ischaemic damage, the consequent oxidative stress damage or the combination of both. Consequently, a wide range of cardiomyopathies, liver and renal failure, and acute brain damage due to ischæmic stroke or the long-term consequence as Alzheimer’s disease lead to a high rate of mortality in humans [16–21]. The high sensitivity of these organs to energy stress makes it a vital requirement for their cells to recruit an immediate strategy for a constant and rapid monitoring of energy level by their metabolic regulators. Here we examined the immediate reaction of LKB1/AMPK/mTOR central metabolic axis, in response to a global ischaemia in these organs. By using a reversible model of cardiac arrest in rat, which was developed in our laboratory [22], we investigated the time-dependent phosphorylation level of LKB1 (Ser^431^), AMPK (Thr^172^) and mTOR (Ser^2448^) along with their non-phosphorylated forms (total proteins) in brain, heart, liver and kidney. The energy situation, presented by ADP/ATP ratio at any time point of ischaemia was also assessed separately in these tissues, in order to study the involvement of ATP levels, as the possible mechanism in changing LKB1/AMPK/mTOR activation under ischaemia.

## Methods

### Animal experiments

The animal experiments in this study were approved by the Animal Ethic Committee of Flinders University. The study was completed in accordance with the South Australian Prevention of Cruelty to Animals Act 1985 following the Australian Code of Practice for the Care and Use of Animals for Scientific Purposes, 2004.

### Animal preparation

Forty-two Sprague-Dawley rats were supplied by Laboratory Animal Services of the University of Adelaide. The animals were randomly divided into 7 groups (1 control: anesthesia only, 6 ischaemic groups, *n* = 6 in each). The rats were kept in Flinders University Animal Facility with free access to food and water until they reached the minimum age of 3 months and the body weight of 250-350 g. Twelve hours before experiments, the rats were fasted with free access to water. On the day of the experiment, anesthesia was initiated by intraperitoneal injection of Ketamine (Sigma, 343,099) and Xylazine (Sigma, X1251), 100 mg/kg and 10 mg/kg body weight, respectively. The tail vein was cannulated using a 22G 0.90 mm intravenous catheter for drug and volume (Saline) delivery. The chest was shaved to provide a clear area for defibrillator electrodes’ attachment and the electrocardiogram was recorded constantly via chest leads using a defibrillator/monitor (Philips HeartStart MRX, Philips Healthcare INC, USA). Oxygen saturation and pulse rate were monitored constantly and were recorded every 5 min via a Pulse-oximeter attached to the animal’s paw. Ventilation was performed via endotracheal intubation using a 16-gauge cannula inserted in the trachea and connected to a specific volume-controlled small animal ventilator with supplemental oxygen at 70 bpm and tidal volume adjusted to 6 mL/kg. Cardiac arrest (CA) was achieved using two phases of transoesophageal alternating current (AC) as previously described (17). Briefly, a pacing catheter (5F) with two end ring electrodes and a 0.5 cm gap was inserted into the oesophagus to a depth of 6–6.5 cm and connected to a current generator ensuring that the current was applied close to the heart without generating irreversible respiratory muscle paralysis. Two phase electrical stimulation using AC current consisting of 50 Hz AC 24 V (phase 1), followed by 50 Hz AC 18 V (phase 2) in order to generate the least oesophageal thermal injury. Ventilation was stopped during the period of CA. CA was confirmed through the electrocardiogram monitor, showing high voltage AC current and a loss of pulse detected by the oximeter within a few seconds after applying the current. Different durations of current (15 and 30 s and 1, 2, 4 and 8 min) caused CA for the relative periods. To obtain brain, heart, liver and kidney samples at the end of CA periods, the animal was decapitated under general anesthesia and the required tissues were immersed immediately in liquid nitrogen and kept in – 80 °C freezer until further analysis.

### Antibodies

Mouse monoclonal phospho-liver kinase b1 (LKB1) (Ser^431^; sc-271,924), rabbit polyclonal LKB1 (H-75; sc-28,788), rabbit polyclonal phospho-adenosine monophosphate kinase protein kinase (AMPK) (Thr^172^; #2531), rabbit polyclonal AMPK (#2532), rabbit polyclonal phospho-mammalian target of rapamycin (mTOR) (Ser^2448^; #2971) and rabbit-polyclonal mTOR (#2972) antibodies were purchased from Cell Signalling. Mouse monoclonal beta actin (ab6276) antibody was purchased from Abcam, USA. Secondary antibodies were purchase from Jackson Immuno Research, USA (HRP donkey anti- mouse and anti-rabbit).

### Tissue homogenates for western blot

The middle 1/3 of the frozen brain containing left parietal cortex and hippocampus, left heart ventricle, middle 1/3 of left kidney containing cortex, medulla, and pelvis, and the left lateral lobe of liver were homogenized in homogenizing extraction buffer containing protease inhibitors of Pepstatin A (Sigma, P5318, 1 μg/ml) and Leupeptin (Sigma, L2884, 1 μg/ml). The homogenate was centrifuged at 1000×*g* for 5 min at 4 °C and the supernatants were stored at − 80 °C until analysed.

### Protein quantification

By using an EZQ assay kit (BioRad, Hercules, CA), the total protein in each sample was measured following the manufacturer’s protocol. Briefly, 10 μL of sample, 25 μL of 4 times sample buffer (100% glycerol, 1 M Tris/HCl pH 6.8, SDS, beta-mercaptoethanol, H_2_O) and 65 μL H_2_O were combined. Ten μL of this solution was added to 90 μL of H_2_O. One μL of samples and standard solution (serial dilutions of ovalbumin) were loaded on assay paper in triplicate each in 96-well plates and absorbance was measured using an Image Master VDS-CL (Amersham Biosciences) and quantified by CareStream molecular imaging software.

### Western blot analysis

To analyse electrophoretic mobility of p-LKB1 (Ser^431^), LKB1, p-AMPK (Thr^172^), AMPK, p-mTOR (Ser^2448^) and mTOR, 30 μg of each sample in sample buffer was loaded to each well of Any kD™ TGX Stain-free gel (Bio-Rad, 569,033), along with 1 well of 5 μL Precision Plus Protein™ Dual Color Standards (Biorad, Hercules, CA, USA). The current (100 V, 300 mA) was applied to the gel for 20 min, to separate the proteins based on their molecular weights. After standard SDS-PAGE separation, the proteins were transferred onto Polyvinylidene Difluoride (PVDF) membrane at 100 V for 30 min. Following electroblotting, the membranes were blocked for 1 h at room temperature in a solution of 5% non-fat dry milk in Tris-buffered saline containing 0.1% Tween 20 (pH 7.6). The separate membranes were incubated overnight at 4 °C with primary antibodies of mouse monoclonal p-LKB1 at Ser^431^ (1:500), rabbit polyclonal LKB1 (1:500), rabbit polyclonal p-AMPK at Thr^172^ (1:1000), rabbit polyclonal AMPK (1:1000), rabbit polyclonal p-mTOR at Ser^2448^ (1:500) and rabbit polyclonal mTOR (1:500). The membrane were incubated (1 h) with the HRP secondary antibodies (donkey anti mouse, 1:3000; donkey anti rabbit, 1:1000) on following day. The blots were then developed using an ECL and the chemiluminescence signal detection was performed using Fuji LAS4000 imager and quantified by CareStream molecular imaging software, and were corrected by actin levels.

### ADP/ATP ratio measurement

The tissue ratio of ADP/ATP was measured via bioluminescent detection of ADP and ATP levels using ApoSENSOR bioluminescent assay kit (BioVision) following manufacturer’s instruction. Briefly, the tissues were homogenized in 6% trichloroacetic acid (TCA) for 1 min followed by 5 min centrifuge at 6000 *g*. After collecting supernatant, TCA was neutralized by tris-acetate. Standard curves were produced with known levels of ADP and ATP and the background luminescence was measured using a Wallac Victor2 1420 multi-label counter. The total ATP was quantified by the addition of the reaction mixture, containing luciferase and luciferin. ATP present in the samples is utilized for the luciferase-catalyzed conversion of luciferin to oxyluciferin. The production of light was quantified as value A by measuring the luminescence, immediately followed by the addition of an ADP-converting enzyme that converts intracellular ADP to ATP and measurement of luminescence (value B). The second luminescence value (value B) represents light generated by total ADP and ATP present in the reaction mixture. After correcting for background luminescence, value A was subtracted from value B to calculate light generated by ADP alone, and ADP/ATP ratio was calculated.

### Statistical analysis

All of the data in the current study were analysed using IBM SPSS Statistics version of SPSS Software. The data are expressed as the mean ± SD. One-way ANOVAs was used to assess the differences between the means of the groups followed by post hoc Tukey’s. Significance was defined as *p* < 0.01.

## Results

### Recording cardiac activity by electrocardiograph (ECG) during cardiac arrest (CA)

**Fig. 1:**
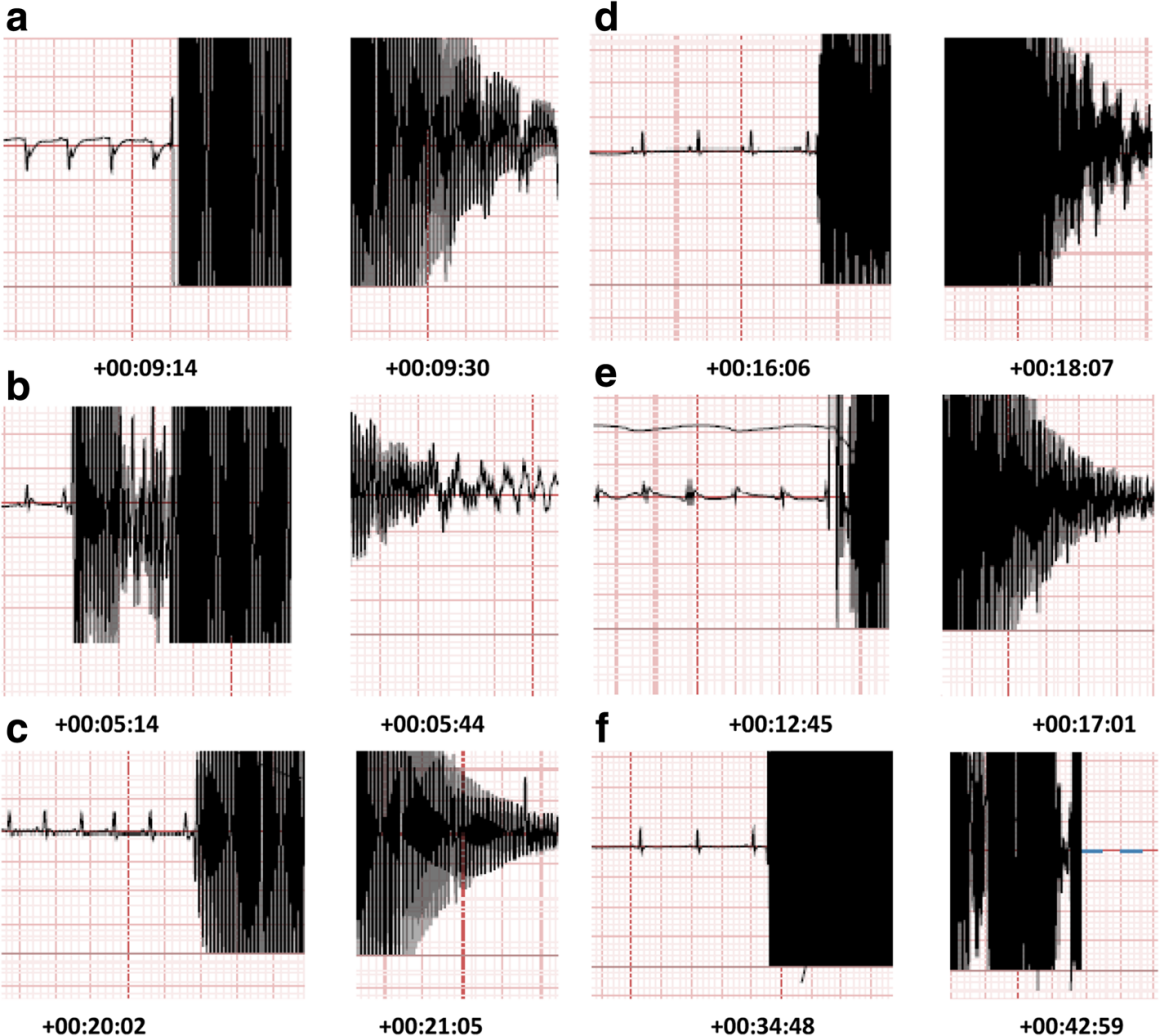
Electrocardiograms of six durations of global ischaemia generated by trans-oesophagus AC current, confirmed by cardiac arrest (CA). **a** 15 s of CA **b** 30 s of CA **c**1 min of CA **d** 2 min of CA **e** 4 min of CA **f** 8 min of CA

To produce a global ischaemia, cardiac arrest was generated by applying 2 phases of trans-oesophageal AC current (50 Hz, 24 V followed by 50 Hz, 18 V) under general anaesthesia, while ECG was recorded via attached electrodes to the rat’s chest. The EEG records showed no heart rhythm during duration of 15 s (Fig. 1a), 30 s (Fig. 1b), 1 min (Fig. 1c), 2 min (Fig. 1d), 4 min (Fig. 1e) and 8 min (Fig. 1f) of applying AC current, confirming CA.

### ADP/ATP ratio in brain, heart, kidney and liver during in ischaemic and control groups

**Fig. 2:**
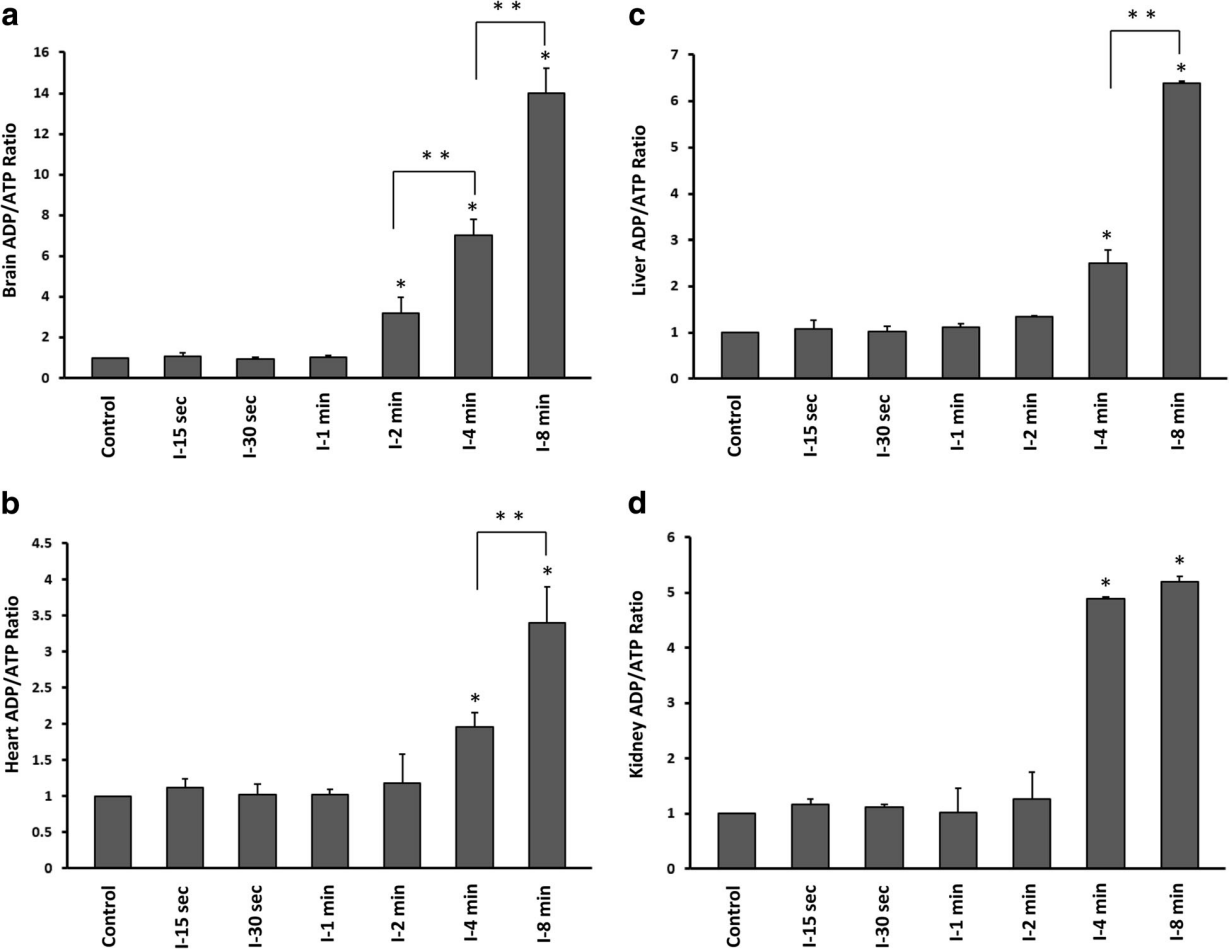
ADP/ATP ratio in six groups of time-dependent ischaemia and control group (anaesthesia only). **a** Brain **b** Heart **c** Liver **d** Kidney. I-15 s: 15 s ischemia, I-30 s: 30 s ischemia, I-1 min: 1 min ischemia, I-2 min: 2 min ischemia, I-4 min: 4 min ischemia, I-8 min: 8 min ischemia *p < 0.001 all groups vs control (One Way ANOVA, followed by post hoc Tukey’s). **p < 0.01 I-8 min vs I-4 min and I-4 min vs I-2 min ischaemia (One Way ANOVA followed by post hoc Tukey’s). Error bars depict the SD

To determine whether short episodes of ischaemia (15 s up to 8 min) affects cellular energy levels, we quantified ADP and ATP concentration and measured the ADP/ATP ratio in brain, heart, liver and kidney of control and ischaemic groups. ADP/ATP ratio was significantly increased in brain following 2 min ischaemia (One Way ANOVA, followed by TUKEY HSD, *p < 0.001, vs control group) in a time-dependent manner (One Way ANOVA, followed by TUKEY HSD, **p < 0.01, 4 min vs 2 min, and 8 min vs 4 min ischaemia) (Fig. 2a, e). In other organs ADP/ATP ratio was enhanced significantly after 4 min ischaemia (One Way ANOVA, followed by TUKEY HSD, *p < 0.001, vs control group) (Fig. 2b-d, e), which was time-dependent in heart and liver (One Way ANOVA, followed by TUKEY HSD, **p < 0.01, 8 min vs 4 min ischaemia) (Fig. 2b, c, e).

### Effect of global ischemia on p-LKB1 (Ser^431^) /LKB1 in brain, heart, liver and kidney

**Fig. 3:**
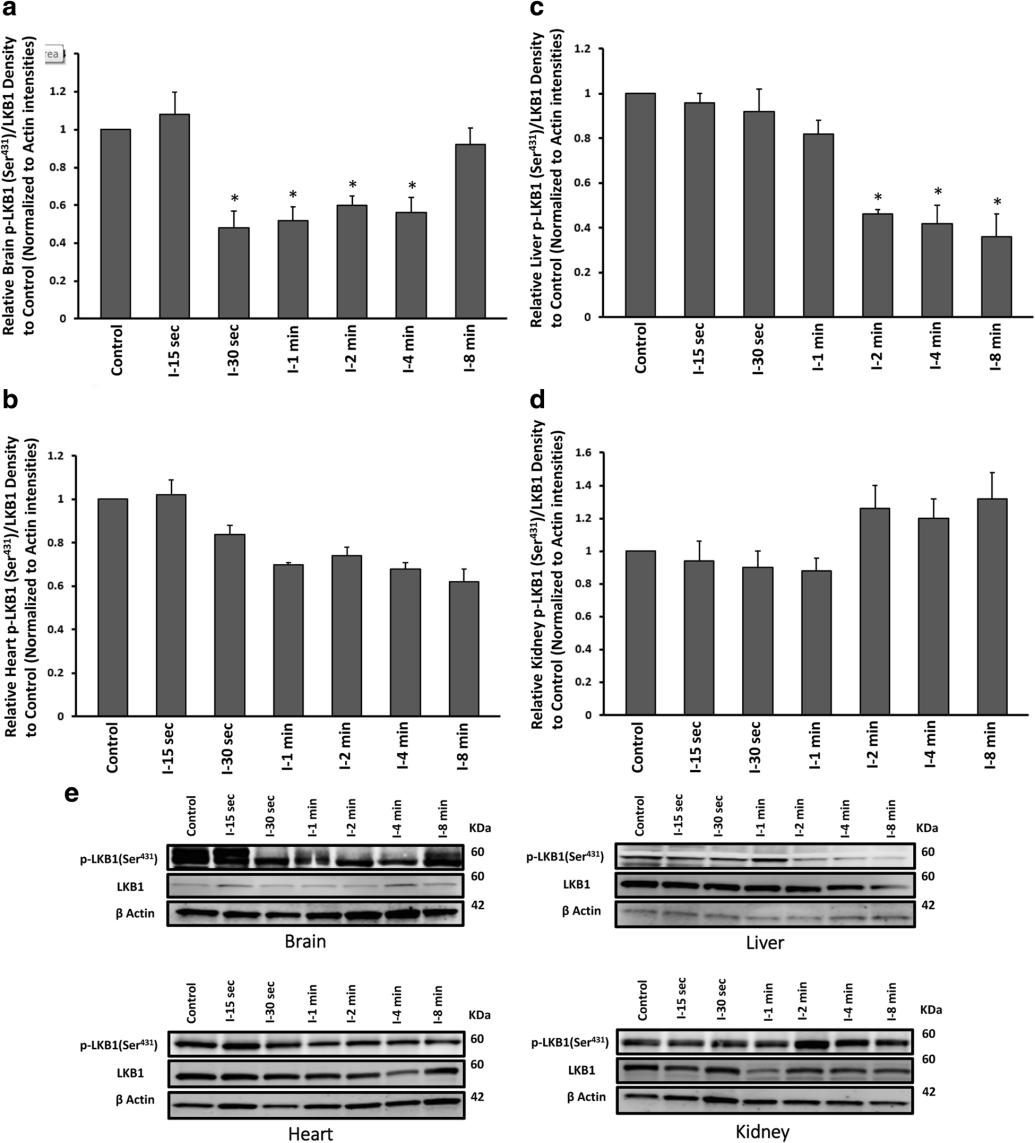
Western blot analysis results of p-LKB1at Ser^431^ and total protein of LKB1 assessment from **a** brain **b** heart **c** liver and **d** kidney tissues of ischaemic and control groups. All values are expressed as percent change relative to control group and were corrected by the Actin level. **e** Protein band intensities of p-LKB1 (Ser^431^), LKB1 and Actin. I-15 s: 15 s ischemia, I-30 s: 30 s ischemia, I-1 min: 1 min ischemia, I-2 min: 2 min ischemia, I-4 min: 4 min ischemia, I-8 min: 8 min ischemia. *p < 0.001 all groups vs control (One Way ANOVA, followed by post hoc Tukey’s). Error bars depict the SD

Previous studied showed the major role for LKB1 as an upstream kinase to facilitate AMPK phosphorylation in response to energy stress [6, 7]. To investigate and compare the activation level of LKB1 (p-LKB1 (Ser^431^)/LKB1 ratio) under ischaemia in our experimental organs, western blot analyses were carried out using left parietal cortex and subcortical hippocampus, left heart ventricle, left middle area of cortex, medulla and pelvis, and the left lateral lobe of liver homogenates of ischaemic and control (anesthesia only) groups. A significant dephosphorylation of LKB1 (Ser^431^) was observed in brain after 30 s ischaemia up to 4 min (One Way ANOVA, followed by TUKEY HSD, *p < 0.001, vs control group), followed by a recurrent rephosphorylation after 8 min of ischaemia (Fig. 3a, e). The similar dephosphorylation of LKB1 (Ser^431^) was seen in liver following 2 min while dephosphorylation was continued to 8 min of ischaemia (One Way ANOVA, followed by TUKEY HSD, *p < 0.001, vs control group) (Fig. 3c, e). LKB1 showed some levels of dephosphorylation at Ser^431^ after 30 s up to 8 min in heart tissue, however the difference was not significant (Fig. 3b, e). In liver, 2, 4 and 8 min of ischaemia, caused some LKB1 hyperphosphorylation at Ser^431^, but the difference was not significant from control group (Fig. 3d, e). In all groups, β-actin levels remained the same, reflected equal loading across all lanes. All values were corrected by actin densities.

### Effect of global ischemia on p-AMPK (Thr^172^) /AMPK in brain, heart, liver and kidney

**Fig. 4:**
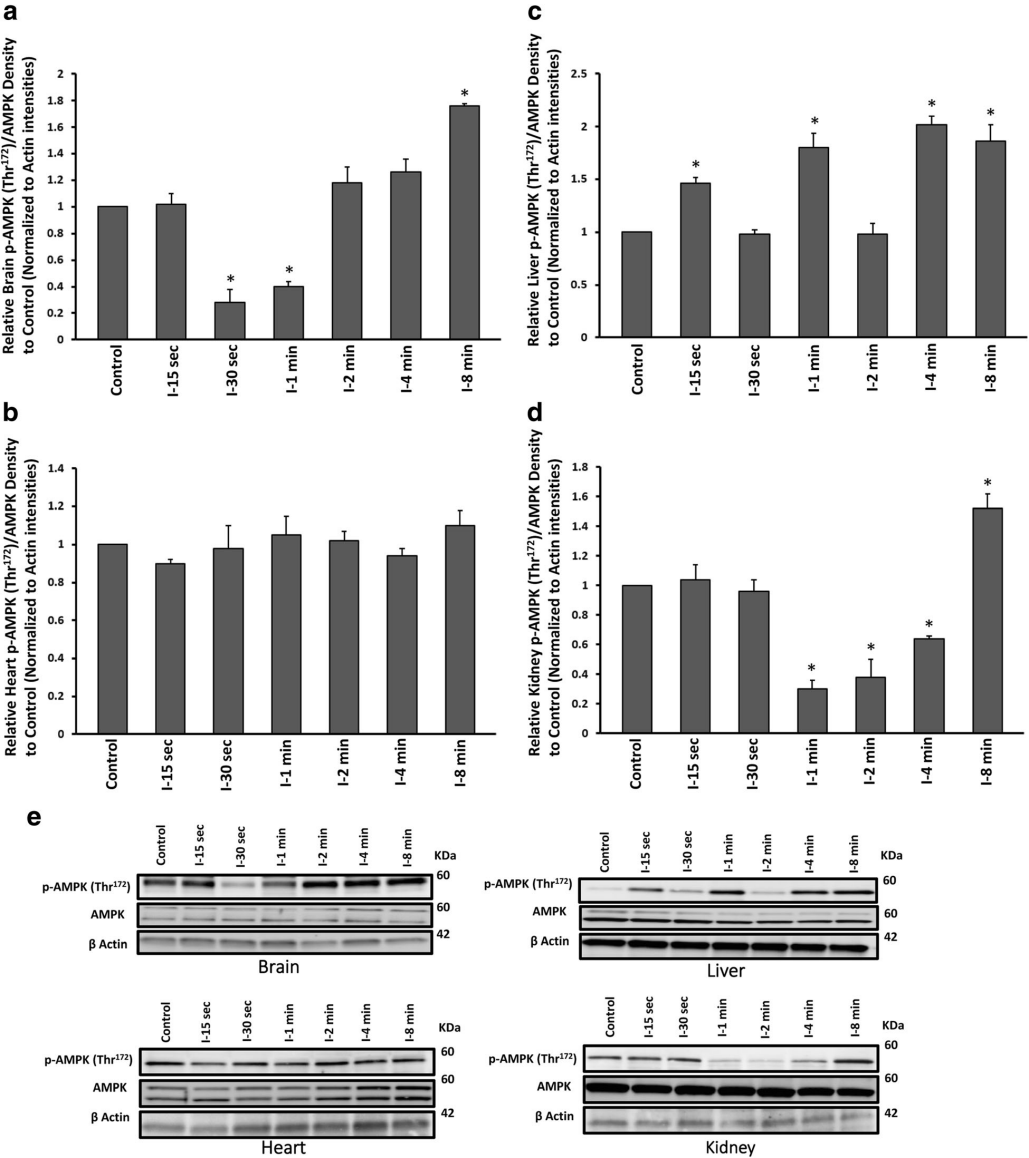
Western blot analysis results of p-AMPK at Thr^172^ and total protein of AMPK assessment from **a** brain **b** heart **c** liver and **d** kidney tissues of ischaemic and control groups. All values are expressed as percent change relative to control group and were corrected by the Actin level. **e** Protein band intensities of p-AMPK (Thr^172^), AMPK and Actin. I-15 s: 15 s ischemia, I-30 s: 30 s ischemia, I-1 min: 1 min ischemia, I-2 min: 2 min ischemia, I-4 min: 4 min ischemia, I-8 min: 8 min ischemia *p < 0.001 all groups vs control (One Way ANOVA, followed by post hoc Tukey’s). Error bars depict the SD

We examined the level of phosphorylated AMPK (p-AMPK) at Thr^172^ and the total AMPK to evaluate and compare the level of AMPK activation as the immediate regulator of cell energy in brain, heart, liver and kidney under time-dependent global ischaemia. AMPK dephosphorylated (Thr^172^) significantly after 30 s and dephosphorylation continued up to 1 min (One Way ANOVA, followed by TUKEY HSD, *p < 0.001, vs control group). Rephosphorylation of AMPK (Thr^172^) occurred after 2 min and became substantially higher than control group after 8 min of ischaemia (One Way ANOVA, followed by TUKEY HSD, *p < 0.001, vs control group) (Fig. 4a, e). A similar pattern of AMPK dephosphorylation at Thr^172^ was observed in kidney, starting after 1 min, when significant rephosphorylation at Thr^172^ occurred after 8 min of ischaemia (One Way ANOVA, followed by TUKEY HSD, *p < 0.001, vs control group) (Fig. 4d, e). AMPK activity (p-AMPK (Thr^172^)/AMPK) was not changed significantly in heart of ischaemic groups (Fig. 4b, e). AMPK showed alternative hyperphosphorylation at Thr^172^ in ischaemic liver tissue in 15 s, 1, 4 min, which continued to 8 min (Fig. 4c, e). In all groups, β-actin levels remained the same, reflected equal loading across all lanes. All values were corrected by actin densities.

### Effect of global ischemia on p-mTOR (Ser^2448^) / mTOR in brain, heart, liver and kidney

**Fig. 5:**
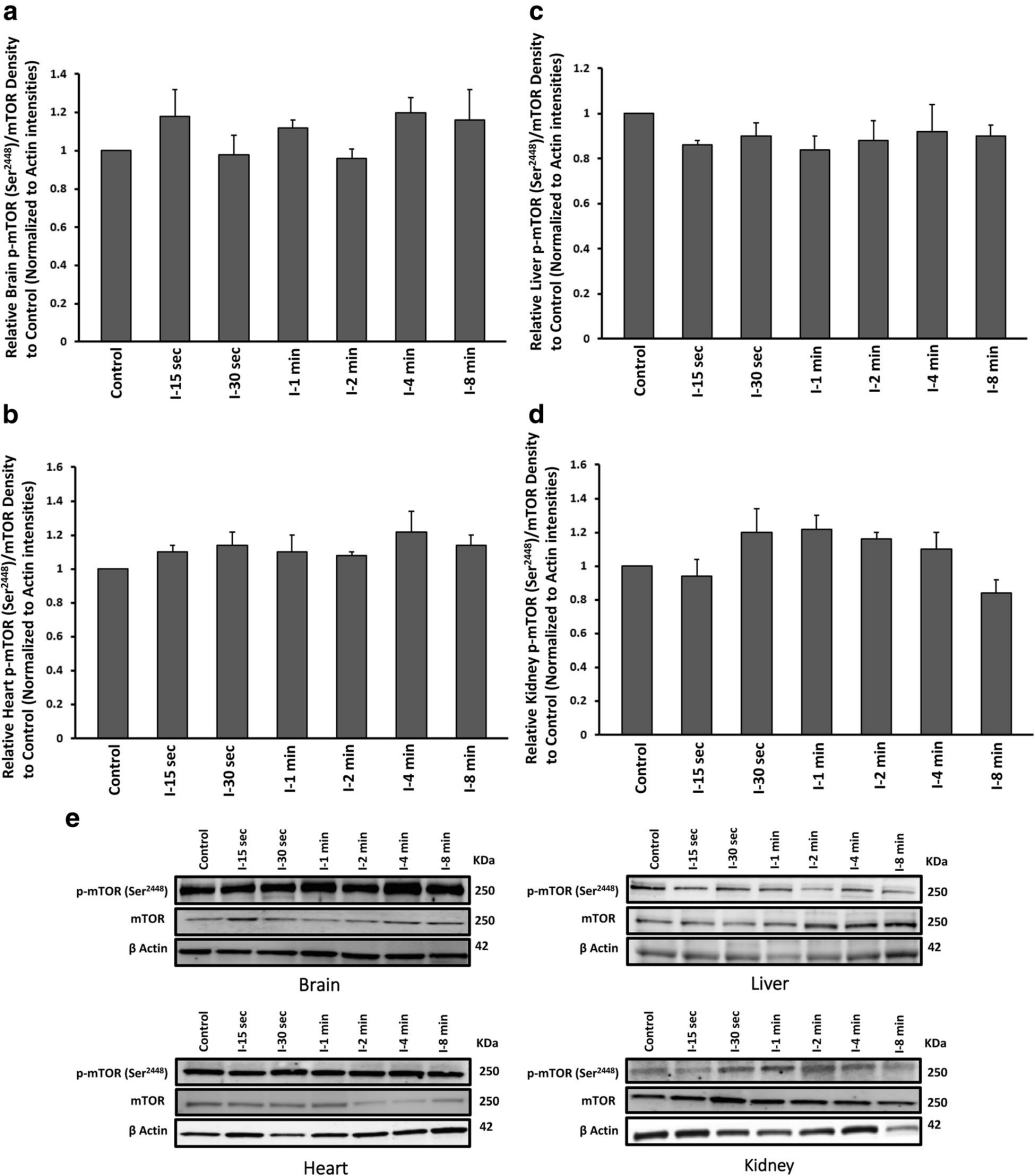
Western blot analysis results of p-mTOR at Ser^2448^ and total protein of mTOR assessment from **a** brain **b** heart **c** liver and **d** kidney tissues of ischaemic and control groups. All values are expressed as percent change relative to control group and were corrected by the Actin level. **e** Protein band intensities of p-mTOR (Ser^2448^), mTOR and Actin. I-15 s: 15 s ischemia, I-30 s: 30 s ischemia, I-1 min: 1 min ischemia, I-2 min: 2 min ischemia, I-4 min: 4 min ischemia, I-8 min: 8 min ischemia *p < 0.001 all groups vs control (One Way ANOVA, followed by post hoc Tukey’s). Error bars depict the SD

The role of mTOR signalling pathway in ischemic disease have been suggested recently [23]. To address any early alteration of mTOR activity during short ischaemic events in four major high metabolic rate organs, the ratio of p-mTOR at Ser^2448^ (active form) to total mTOR was evaluated. In kidney tissue, mTOR showed some levels of phosphorylation (Ser^2448^) after 30 s to 4 min ischaemia, however the difference was not significant. In all other tissues, mTOR activity (p-mTOR (Ser^2448^)/mTOR) was remained unchanged in ischaemic groups compare to control (Fig. 5a-e). In all groups, β-actin levels remained the same, reflected equal loading across all lanes. All values were corrected by actin densities.

## Discussion

The current study revealed a range of various early strategies of brain, heart, kidney and liver in activating liver kinase B1 (LKB1), adenosine monophosphate protein kinase protein (AMPK) and mammalian target of rapamycin (mTOR) signalling pathways in response to ischaemia. To our knowledge, this the first time that LKB1/AMPK/mTOR immediate ischaemic response of high metabolic rate tissues have been studied in a very short window of time (15, 30 s and 1, 2 min of ischaemia). These major solid organs have the highest rates of metabolism and consequently the highest sensitivity to energy stress. There are numerous studies, reporting that ischaemic injuries to brain, heart, kidney and liver are leading causes of mortality and disability around the world [16, 17, 24–26]. It is generally agreed that the first immediate protective reaction against ischaemia is mediated by a complex cellular metabolic pathway of LKB1/AMPK/mTOR in almost all tissues. AMPK plays a central role in this axis as the master of energy regulators [5, 6]. Previous studies showed the enhanced level of AMPK phosphorylation (Thr^172^) in response to energy stress, especially when the situation persisted [2, 27]. Our results demonstrated a high level of AMPK activation, particularly in brain, kidney and liver, especially in the presence of persistent ischaemia up to 8 min. Consistent with the studies on longer period of hypoxia, AMPK hyperphosphorylation (Thr^172^) in our study, was associated with a substantial reduction in ATP levels (a significant increase in ADP/ATP), which was reached to its maximum decline at 8 min.

ATP drop down, along with increasing in production of reactive oxygen spices (ROS) are from the major activators of AMPK [11, 14]. Here, the ATP level was the same as control up to 1 min, for the brain, and up to 2 min for the other tissues in our study. We showed the first significant brain ATP decline after 2 min, while for the other organs it did not reduce before 4 min. Winn et al. [28] previously reported that in brain, the level of ATP reduced to half within the first minute of ischaemia, while AMP elevated to 10 times and ADP doubled. In our study, however, the initial significant change in brain ADP/ATP was observed after 2 min. We believe this difference is due to our improved system of brain isolation, based on an immediate immersing of the skull and the brain (same as the other organs) in liquid nitrogen. Under this condition, the tissues had zero time to consume any ATP during tissue isolation process, which could cause a substantial difference when it comes to an accurate evaluation of ADP to ATP ratio.

It was previously reported that decreasing cellular ATP (an increase in ADP and consequently AMP/ATP ratio), increased AMP binding to AMPK α-subunit, leading to AMPK phosphorylation at Thr^172^ residue and its activation. Activated AMPK switches off biosynthetic pathways that consume ATP while it switches on the one that generate more ATP. Our findings, showing a substantial decrease in ATP in longer periods of ischaemia, especially after 8 min could explain our other observation of AMPK hyperphosphorylation (Thr^172^) in brain and kidney, while these organs also presented the higher sensitivity in reacting to ischaemia, compare to other tissues.

The phosphorylation of AMPK is facilitated by LKB1, the other member of this metabolic axis. This AMPK upstream serine/threonine kinase [8, 10, 11] facilitates AMPK phosphorylation at Thr^172^ in an ATP-dependent [5, 6, 10] and ATP-independent manner [14, 29], with its Ser^428^ phosphorylation (equals to Ser^431^ in rodents) is essential for AMPK activation [30]. Despite what we hypothesized, the immediate impact of ischaemia on LKB1/AMPK axis was not their activation in this study. Instead of hyperphosphorylation, AMPK was subjected to a significant dephosphorylation at Thr^172^ after 30 s and 1 min ischaemia, while the ratio of ADP/ATP remained unchanged. This finding supports the previous reports, including ours, showing an immediate dephosphorylation of some cellular peptides such as tau and AMPK, rather than their hyperphosphorylation, in response to hypoxic situations in brain [31–33]. Not only AMPK, but also LKB1 was dephosphorylated (Ser^431^) in brain and liver to significant levels, and in heart but not to significant levels. LKB1 is constitutively phosphorylated at Ser^431^ [34, 35], and its dephosphorylation, along with AMPK dephosphorylation at Thr^172^ could be considered as an immediate response to ischaemia. This is consistent with previous reports, suggesting protein dephosphorylation as an immediate response to ischaemia [32, 33], and possibly in order to save valuable energy by avoiding making phosphate bonds under energy stress.

As the ADP/ATP levels did not show a dramatic change in 15 and 30 s and 1 min ischaemic brains, and in 15 and 30 s and 1 and 2 min in other tissues in our study, we suggest that the observed dephosphorylation of AMPK (Thr^172^) and LKB1 (Ser^431^) were ATP-independent events. AMPK dephosphorylation (Thr^172^), however was affected possibly by LKB1 dephosphorylation (Ser^431^), regardless of cell ATP levels. Longer periods of ischaemia up to 4 min caused AMPK rephosphorylation (Thr^172^) in brain, and it reached to a significant hyperphosphorylation after 8 min in our study. Rephosphorylation and hyperphosphorylation of AMPK in our brain samples occurred in parallel with a significant drop down in ATP (showing by ADP/ATP increase), which is consistent with the expected effect of energy stress on AMPK [5, 6]. Rephosphorylation of LKB1 however occurred with a delay, staring at 8 min ischaemia. It supports our hypothesis, suggesting that alteration in AMPK phosphorylation during short episodes of ischaemia is an ATP-independent phenomenon, but could be a consequence of LKB1dephosphorylation in brain. Our results also suggest that during longer periods of ischaemia and a dramatic decrease in ATP, AMPK phosphorylation was not affected by p-LKB1 status. As AMPK could also be activated in a Ca^2+^/calmodulin-dependent protein kinase kinase β (CaMKKβ)-dependent but LKB1-independent way [29], this observed finding in our study could be a consequence of such an activation, although it requires future studies to confirm.

Our data demonstrated that the first reaction of both LKB1 and AMPK in brain and kidney is dephosphorylation, rather than hyperphosphorylation. Either this dephosphorylation works as a backup to refill the cellular energy source or simply occurs to save the cells from spending the valuable energy on phosphate bonds to phosphorylate proteins, requires further investigations.

Among the investigated tissues in this study, liver was the only one that showed a progressive LKB1 dephosphorylation (Ser^431^) in parallel with liver’s ATP drop down, and not before that. Dephosphorylation of LKB1 (Ser^431^) started with a delay compare to brain, after 2 min and continued to 8 min. On the other hand, AMPK hyperphosphorylation (Thr^172^) started quite early after 15 s of ischaemia in liver and was repeated in an alternative pattern of relapse of phosphorylation in 30 s and 2 min with hyperphosphorylation (Thr^172^) in between (1, 4 and 8 min ischaemia). Previously, it was shown that ATP concentration remains stable in liver most of times, unless sever hypoxic situation occurs. During an ultimate hypoxic insult, however, hepatic AMPK activation acts as the fundamental strategy to protect cells against hypoxic damage [36]. Our findings showed the same ADP/ATP level in the liver of early (15 s, 30 s, 1 min, 2 min) ischaemic groups as control group while ATP drop down started at 4 min and substantially decreased after 8 min ischaemia. LKB1 dephosphorylation pattern in liver was almost the same as brain, although starting with a delay and continued up to 8 min. That suggests the higher sensitivity of brain LKB1 pathway, in sensing early ischaemia and the later ADP/ATP increase. The pattern of alternative AMPK hyperphosphorylation (Thr^172^) of liver in our study, proposed a possible role for AMPK early activation as immediate strategy to stabilize hepatic ATP levels. Increase in AMPK activation after 15 s ischaemia, followed by returning to baseline phosphorylation after 30 s, and repeating this pattern after 1, 2 and 4 min ischaemia, could justify the observed stable pattern of ADP/ATP ratio in liver in our results, same as previous findings, showing ATP stability of hepatic cells in other studies [36]. Eight min ischaemia, however could be considered as a sever insult, forcing AMPK to remain hyperphosphorylated (Thr^172^), while the significant dropdown in ATP level also persisted.

Our results demonstrated an increase in ADP/ATP ratio in heart and kidney after 4 min ischaemia. Here, kidney showed the higher sensitivity to very short episodes of ischaemia secondary to brain, reaching to maximum increase in ADP/ATP after 4 min ischaemia. We observed some decreased activity of LKB1 (p-LKB1 (Ser^431^)/LKB1) in heart and increased LKB1 activity in kidney, but surprisingly the level of changes were not significant. A significant dephosphrylation of AMPK (Thr^172^) after 1 min ischaemia continued until 4 min, followed by a hyperphosphorylation (Thr^172^) in 8 min in kidneys. It suggested that kidney followed the same pattern as brain in AMPK dephosphorylation and hyperphosphorylation with a small delay in starting dephosphorylation. While previous studies supported our observation of AMPK hyperphosphorylation after 8 min, in longer period of ischaemia (30 and 45 min) [37], the literature’s substantial studies in reporting the immediate reaction of AMPK in kidney in response to ischaemia are very limited.

Despite decreasing ATP after 4 and 8 min ischaemia, AMPK did not show a significant activation (p-AMPK (Thr^172^)/AMPK) in ischaemic hearts up to 8 min. Previous studies showed that 30 min low-flow ischemia increased the activity of α1 and α_2_-subunits of AMPK up to three folds [27], however AMPK activity during shorter episodes of ischaemia have not been investigated. Our results suggest that during short episodes of ischemia, activation of AMPK is not the immediate mechanism of choice for heart in response to ischaemia, at least compare to brain and kidney. One explanation is the simultaneous activation of Akt pathway in the heart under ischaemia, which was reported previously [38]. This activation, in particular in heart, inhibits AMPK phosphorylation [39], although revealing the exact mechanism of this interaction in heart tissue requires further investigation.

Next, we examined the activity of mTOR, as the third element of LKB1/AMPK/mTOR cellular metabolic axis. We observed no substantial difference in mTOR activity level (p-mTOR (Ser^2448^)/mTOR) between ischaemic and control groups in any of our four tested organs. Phosphorylation of AMPK was reported to reduce the activation of mTOR pathway as evidenced by reduced phosphorylation of mTOR on Ser^2448^ [12]. Previous studies indicated enhanced mTOR activity in brains of rats following 3 and 6 h reperfusion after 10 min ischaemia [40]. Matsui et al. [41] reported an increase in AMPK activity with a concurrent decrease in phosphorylated mTOR in heart cell after 2, 6 and 24 h of ischaemia, however there are no results on immediate response of mTOR pathway to very short events of ischaemia, such as what we have examined in the current study. Here, we believe that during ischaemic periods, only immediate strategies of protecting cells could be observed while a significant change in mTOR activity needs longer periods of ischaemic insult. In the other hand, it seems that enhanced level of mTOR activity, which is important in many cellular functions such autophagy and cell proliferation, requires the establishment of damage, and would not act as an energy sensor, in the same way that LKB1 and AMPK work.

## Conclusion

In conclusion, our results indicate that immediate ischemic response of brain and kidney consists a nearly similar pattern of early ATP-independent AMPK dephosphorylation (Thr^172^) followed by a possible ATP-dependent hyperphosphorylation at Thr^172^ (with the higher sensitivity of brain in sensing early ischaemia). This response is different from liver’s AMPK pattern of phosphorylation, which is mostly based on early repeated episodes of hyperphosphorylation (Thr^172^) and relapse (to the baseline phosphorylation level), and eventually an established AMPK hyperphosphorylation (Thr^172^) in longer periods of ischaemia. We hypothesize that liver uses this pattern to minimize cellular ATP changes, however additional studies are necessary to confirm this hypothesis. Finally the possible higher involvement of Akt pathway in the heart with its inhibitory action on AMPK, could explain the heart minimum changes of immediate LKB1/AMPK activity in response to ischemia among all four organs. Further investigations are required to reveal the other underlying mechanisms such as cellular levels of calcium and ROS generation in creating these differences in ischaemic response between high metabolic rate tissues.
